# Influence of In Vitro Digestion on Antioxidant Activity of Enriched Apple Snacks with Grape Juice

**DOI:** 10.3390/foods9111681

**Published:** 2020-11-17

**Authors:** Constanza Pavez-Guajardo, Sandra R. S. Ferreira, Simone Mazzutti, María Estuardo Guerra-Valle, Guido Sáez-Trautmann, Jorge Moreno

**Affiliations:** 1 Food Engineering Department, Universidad del Bío-Bío, Casilla 447, Chillán 4081112, Chile; constanza.pavez.g@gmail.com (C.P.-G.); maria.guerra1601@egresados.ubiobio.cl (M.E.G.-V.); gusaez@egresados.ubiobio.cl (G.S.-T.); 2 Chemical and Food Engineering Department, Federal University of Santa Catarina, Florianópolis 88040-900, Brazil; s.ferreira@ufsc.br (S.R.S.F.); monemazzutti@gmail.com (S.M.)

**Keywords:** enriched apple snack, in vitro digestion, antioxidant activity

## Abstract

Fruits are sources of bioactive compounds (BACs), such as polyphenols. This research aimed to study the in vitro bioaccessibility of polyphenols from enriched apple snacks with grape juice and determine their antioxidant capacity. Impregnation (I) treatments were carried out at atmospheric pressure and in a vacuum (IV) at 30, 40, and 50 °C and their combinations with ohmic heating (OH), I/OH, and IV/OH. Later, samples were dehydrated by forced convection at 40, 50, and 60 °C. Enriched samples were subjected to in vitro digestion. The total polyphenols, monomeric polyphenols, and antioxidant activities were determined from recovered extracts. Results showed that total polyphenols present in higher concentrations in the gastric phase, 271.85 ± 7.64 mg GAE/100 g d.m. Monomeric polyphenols’ behavior during in vitro digestion for the VI/OH 50 °C and dried treatment (60 °C) was descending, mainly in quercetin, which decreased by 49.38% concerning the initial concentration, before digestion. The cyanin, catechin, epicatechin, and epigallocatechin decreased by 26.66%, 20.71%, 23.38%, and 21.73%, respectively. Therefore, based on obtained results, the IV/OH 50 °C treatment (dried 60 °C) is the best combination to incorporate polyphenols from grape juice.

## 1. Introduction

To achieve products with health-promoting characteristics, food enrichment strategies, such as vacuum impregnation [1], vacuum ultrasonication [2], ultrasound-assisted, and osmotic impregnation [3], are widely applied. Another technology used is ohmic heating, which converts electrical into thermal energy, allowing an even and rapid increase, resulting in a more effective process guaranteeing nutrient retention [4]. Antioxidants’ effects depend on their concentrations in fruits and vegetables and their bioaccessibility and bioavailability after ingestion. In vitro digestion has often been used to simulate gastrointestinal conditions because it can be considered relatively simple when compared to in vivo models in addition to being safe and not presenting ethical restrictions [5].

Bioactive compounds enter the organism through digestion, allowing for the extraction of macronutrients (e.g., lipids) and micronutrients (e.g., minerals) from the food matrix into humans [6]. In vitro digestion allows us to determine those nutrients’ bioaccessibility once the food is ingested [7]. This bioaccessibility assay is generally divided into three phases for better understanding: the mouth, gastric, and intestinal phases, which use α-amylase, pepsin, and a mixture of bile salts and pancreatin, respectively [8], to release the bioactive compounds.

Many studies have established the importance of polyphenols’ bioaccessibility in grapes [9] and apples [10]. However, few studies have evaluated the antioxidant characteristics of apple snacks enriched with grape juice using vacuum impregnation (VI) and ohmic heating (OH) under in vitro simulated gastric conditions; some similar studies have been reported by Barrera et al. [11]. These technologies have been applied to incorporate folic acid and arginine using apples as a matrix and probiotics into dry fruit without studying their bioaccessibility [1,12,13]. The main flavanols present in grape juices are procyanidins, epicatechin, and epigallocatechin, which have anti-inflammatory and antimicrobial properties [14]. Among the monomeric polyphenols in apples are gallic acid, procyanidin B1, catechin, procyanidin B2, epicatechin, syringic acid, and quercitrin [15]. Polyphenols are hydrolyzed either by enzymes or gut microbiota metabolized; consequently, these compounds differ from those present in food. Once biotransformed, they are low molecular weight compounds. Therefore, they are more bioavailable [16], potentiating its beneficial effects on health. The food matrix and the amount ingested may also influence antioxidants’ bioavailability of antioxidants [16]. It should be mentioned that only 5–10% of phenolic compounds, despite having high bioavailability, are absorbed in the small intestine, while a larger percentage (90–95%) is metabolized in the large intestine native microbiota [16]. Thus, this study aimed to investigate the effects of simulated gastrointestinal digestion of enriched apple snacks on the recovery of total polyphenols and monomeric polyphenols by HPLC. Additionally, antioxidant activity was assessed before and after enriched apple snack digestion.

## 2. Materials and Methods

Raw material and chemical reagents.

The raw material, apple (cv. Fuji), and grape (cv. País) were collected from the local market (Chillán, Chile 2019). The chemical reagents were used for the different analyses: NaHCO_3_, NaCl, KCl CaCl_2_·2 H_2_ O, K_2_ HPO_4_, HCl, Folin-Ciocalteu, gallic acid, 1,1-diphenyl-2-picrylhydrazyl (DPPH); 2,4,6-tripyridyl-*s*-triazine, chloroform, anhydrous citric acid were acquired from Merck, Germany. Also, mucin, α-amylase, pepsin, pancreatin, bile salts, delphinidin, caffeic acid, myricetin, quercetin, (-)-epicatechin, (+)-catechin, (-)-epigallocatechin gallato, malvidin, *p*-coumaric acid, cyaniding, Tween-20 were purchased from Sigma-Aldrich, USA. Syringe tip 45 filters µm PTFE from Merck Millipore, Ireland, were used. Ultrapure water was obtained using a Thermo, Scientific filter.

### 2.1. Sample Preparation

Apple (cv. Fuji) and grape (cv. País) were acquired from local markets (Chillán, Chile 2019). The fresh raw materials were stored under refrigeration (5 °C) until processing (maximum 48 h). Apples were peeled manually and cut into 5-mm-thick slices (57 mm external and 17 mm internal diameter) [1]. Grape juice was obtained using a juice processor (NEX, J-2000). Sample preparation was carried out at the Laboratory of Analysis and Minimum Fruit Processing of the University of Bío-Bío, Chile.

### 2.2. Enriched Apple

Enriched apple slices were obtained by applying impregnation (I), (VI), (OH), and vacuum impregnation combined with ohmic heating (VI/OH) as described by Guerra-Valle et al. [17]. Finally, the enriched apple snacks were generated with a drying process described by Moreno et al. [1] using three temperatures: 40, 50, and 60 °C.

Vacuum impregnation and ohmic heating conditions were as follows: vacuum pressure of 50 mbar/5 min and processed at 40, 50, or 60 °C using a drying oven at an air velocity of 1.5 m/s.

The selected combinations of temperature conditions that provided the best retention of total polyphenols (impregnation temperature/drying temperature) were 50/60 °C for enriched apple snacks impregnated with grape juice. Based on the total polyphenol content, the treatment with the highest retention was selected for in vitro digestion. The fresh apple was used as a control sample for each of the analytical determinations. To report the results of each test per 100 g of dry matter (100 g d.m.), the apple sample humidity was considered, as described by Guerra-Valle et al. [17].

### 2.3. In Vitro Digestion Process

The in vitro digestion process was performed according to Tenore et al. [18] and Poinot et al. [19], with modifications. Artificial saliva was a prepared solution containing NaHCO_3_ (5.21 g/L), NaCl (0.88 g/L), KCl (0.48 g/L), CaCl_2_·2 H_2_ O (0.44 g/L), K_2_ HPO_4_ (1.04 g/L) (Merck, Germany), mucin (2.16 g/L), and α-amylase (13.00 g/L) (Sigma-Aldrich, USA). The pH was adjusted to 6.8, with 0.1 N HCl, and adjusted to a volume of 100 mL with ultrapure water.

For the chewing (mouth) phase, 5 g of sample was weighed and placed in a plastic bag along with 80 mL of ultrapure water, and 12 mL of artificial saliva was added. The mixture was placed in a stomacher for 2 min.

Gastric phase: After the mouth phase, 10 mL of pepsin solution (15 mg/15 mL of ultrapure water) was added, and the pH was adjusted to 2.0 with 6 N HCl. The sample was placed in a thermoregulated bath with stirring at 37 °C for 2 h.

Intestinal phase: The pH of the above solution was adjusted to 6.5 with NaHCO_3_. Ten milliliters of a mixture of pancreatin (60 mg/15 mL) with bile salts (750 mg/15 mL) was added. The sample was placed in a thermoregulated bath with stirring at 37 °C for 2 h. To simulate intestinal conditions, amber colored bottles were used, and these bottles were covered with aluminum foil. A static method was used to simulate the stages of the digestion process. Digestion was divided into five phases: mouth, gastric, and intestinal phases (initial, middle, and final). Samples of the initial intestine were taken at the beginning of intestinal digestion, those of the middle intestine after one hour of intestinal digestion, and the final sample at two hours.

### 2.4. Total Polyphenol Content during the In Vitro Digestion Process

Total polyphenol content was determined using the Folin Ciocalteu method described by Waterhouse [20] using a linear range for the standard curve was between 50 to 500 mg/liter in a spectrophotometer (Genesys 10 S UV-Vis, Thermo Scientific). The results were reported in milligrams of gallic acid equivalents in 100 g of dry matter (mg GAE / 100 g d.m. After each phase of in vitro digestion, 15 mL of sample was taken and centrifuged (Eppendorf Centrifuge 5430 R, Germany) at 4000 RPM and 4 °C for 10 min. The supernatant was filtered (0.45 µm) before performing the analyses [21].

### 2.5. Analysis of Monomeric Polyphenols by HPLC

An aliquot of 3 mL was taken and filtered using 45 µm PTFE syringe tip filters (Merck Millipore, Ireland) into 2 mL vials. The following standards were used, according to availability in the laboratory depending on the investigations carried out: delphinidin, caffeic acid, myricetin, quercetin, (-)-epicatechin, (+)-catechin, (-)-epigallocatechin gallato, malvidin, *p*-coumaric acid, and cyanidin (Sigma-Aldrich, USA). Only the identified standards were reported. The HPLC-DAD instrument (Perkin Elmer, series 200, Massachusetts, USA) consisted of a binary pump, an autosampler, a diode array detector, a column compartment, and a Purospher STAR^®^ 100 RP-18 e columns (125 × 4 mm, 5 μm particle size) as described. Working conditions were established according to Ruiz et al. [22] using the following mobile phases: A= acetonitrile/formic acid/water (3:10:87); B = formic acid/water/acetonitrile (10:40:50), modifying the gradient of mobile phases. The DAD wavelengths were 280 and 520 nm for flavan-3-ols and anthocyanins, respectively.

### 2.6. Antioxidant Capacity in the Snacks during In Vitro Digestion

Antioxidant capacity determinations were carried out at the Laboratory of Thermodynamics and Supercritical Technology (LATESC) from the Chemical and Food Engineering Department at the Federal University of Santa Catarina, Florianópolis, Brazil. As stated above, after each phase of in vitro digestion, 15 mL of supernatant was taken and centrifuged (Eppendorf Centrifuge 5430 R, Germany) at 4000 RPM and 4 °C for 10 min. The supernatant was filtered (0.45 µm) before performing the analyses [17].

#### 2.6.1. DPPH Radical Scavenging Method

The free radical scavenging capacity of the enriched apple snack was determined using 1,1-diphenyl-2-picrylhydrazyl (DPPH), as described by Mensor et al. [23]. Samples were measured at a wavelength of 517 nm with a spectrophotometer (Genesys 10 S UV-Vis, Thermo Scientific). The DPPH was expressed in EC_50_, representing the concentration that decreases 50% of absorbance than a blank (expressed in µg/mL). The EC_50_ results were provided by a linear regression of antioxidant activity curves from each extract concentration.

#### 2.6.2. β-. Carotene Bleaching Method

The *β*-carotene/linoleic acid antioxidant activity of the enriched apple snack extracts was assessed as described by Matthäus [24]. Briefly, a solution of *β-*Carotene in chloroform (0.2 mg/mL, 2 mL) was added to a mixture of linoleic acid (20 μL) and Tween 20 (100 μL), and the chloroform was subsequently removed from the resulting mixture by a vacuum rotary evaporator at 40 °C. Distilled water (100 mL) was then added to the residue, and the resulting mixture was vigorously stirred to form an emulsion. The emulsion (240 μL) was then added to an aliquot of the sample (10 μL), and the absorbance was measured at 460 nm against a blank containing the emulsion without *β*-carotene. The assay mixture was then placed in a water bath at 50 °C, and its absorbance was measured at 0 min and 120 min. The antioxidant activity was then calculated using the following equation:

(1) $$Antioxidant\;activity\;\left(\%\right)=\left(\frac{sample\;A460}{control\;A460}\right)*100$$

To the β-Carotene bleaching assay, a control sample, replacing the extract with water, was prepared, and the antioxidant activity of the extract was calculated. 

#### 2.6.3. Ferric Reducing Antioxidant Power (FRAP) Method

The ferric reducing antioxidant power (FRAP) method was performed as proposed by Benzie and Strain [25], with some modifications by Arnous et al. [26], and was used to determine the antioxidant activity via iron reduction. The reaction mixture was composed of 0.2 mL of extract solutions and 0.2 mL of ferric chloride (3 mM in 5 mM anhydrous citric acid). Blanks were prepared with 0.2 mL of ethanol for analysis instead of an extract. The mixture was agitated and kept at 37 °C for 30 min. Then, 3.6 mL of TPTZ (2,4,6-tripyridyl-*s*-triazine) was added, followed by vortexing and cooling for 10 min to measure the absorbance 620 nm. The extract reducing power was evaluated compared to the Trolox solutions’ standard curve (from 0 to 500 μmol Trolox/extract). The results are expressed as TE (µM/g) and presented as the mean ±standard deviation of triplicate assays.

### 2.7. Statistical Analysis

Data were subjected to analysis of variance (ANOVA) and the LSD test using the statistical program Statgraphics Centurion XVI Software (Statgraphics, Virginia, USA, 2009), with 95% confidence levels (with significance determined by *p* ≤ 0.05). To verify the statistical significance, mean ± standard of three independent measurements.

## 3. Results

### 3.1. Impregnation Kinetics of Polyphenols from Grape Juice

**Figure 1:**
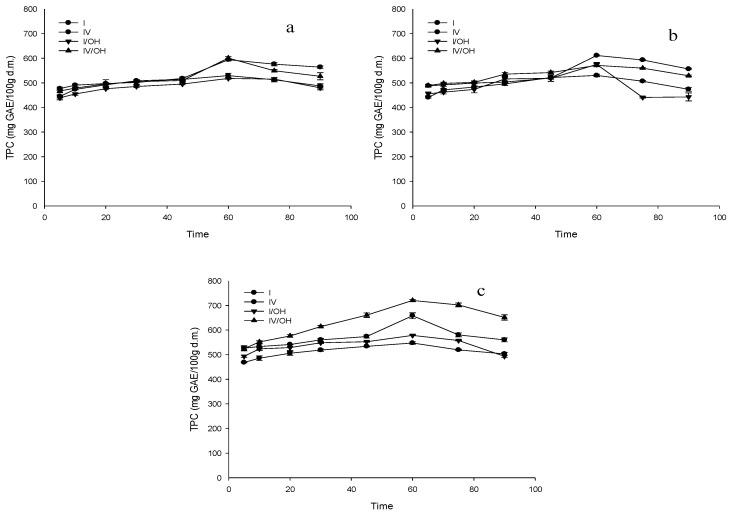
Impregnation kinetics of apple slices with grape juice at three different temperatures, 30 °C (**a**), 40 °C (**b**), and 50 °C (**c**). I: atmospheric pressure and conventional heating; I/OH: atmospheric pressure and ohmic heating; VI: vacuum impregnation and conventional heating; and VI/OH: vacuum impregnation and ohmic heating at 13 V/cm.

To quantity polyphenols from grape juice incorporated into the apple matrix, different impregnation treatments were studied: atmospheric pressure (I), vacuum impregnation (VI), and ohmic heating (I/OH and VI/OH) at temperatures of 30, 40, and 50 °C. These results are those observed in Figure 1. For the three temperatures applied (30, 40, and 50 °C) and impregnation methods studied, the incorporation of bioactive compounds (polyphenols) increased for up to 60 min and then decreased for most of the studied conditions.

### 3.2. Total Polyphenol Content during In Vitro Digestion

**Figure 2:**
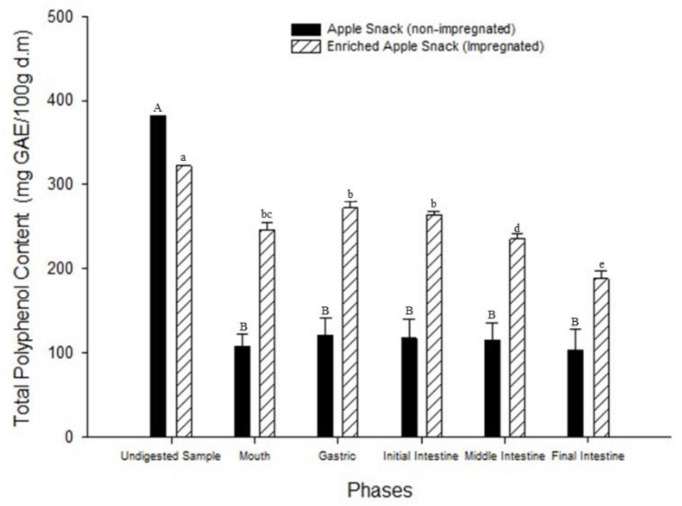
Total polyphenol content (TPC) in apple and enriched apple snacks with grape juice using VI/OH treatment during in vitro bioaccessibility. A, B: Significant differences (*p* ≤ 0.05) in apple snacks (non-impregnated) according to the LSD test were identified. a, b…: Significant differences (*p* ≤ 0.05) in enriched apple snacks (impregnated) according to the LSD test were identified.

Changes that occurred during in vitro digestion of the apple snack (undigested and digested sample) and the apple snack enriched with grape juice (undigested and digested sample) are shown in Figure 2. The total polyphenol concentrations of the undigested apple snack (fresh/dried) and the enriched apple snack (impregnated with grape juice) were 382.3 ± 10.20 mg GAE/100 g d.m. and 322.29 ± 10.60 mg GAE/100 g d.m., respectively. In vitro digestion study was divided into five phases (mouth, gastric, and small intestinal (initial, middle, and final)). For the apple snack (not impregnated), there were no significant differences during in vitro digestion from the mouth phase to the initial intestinal phase. Then, upon completing the digestion process, the phenolic content decreased significantly (Figure 2). For the pair of samples, the control, and enriched apple snack, the phenolic compound concentrations throughout the gastric phase were significantly higher than those in the other phases of in vitro digestion. This behavior is due to phenolic compound hydrolysis when bound to carbohydrates and proteins from the food matrix by enzymatic action and low pH during gastric digestion [27]. In the small intestinal phase (initial, middle, and final intestinal), the phenolic content decreased significantly due to pH changes, from medium acid (gastric digestion) to a medium alkaline [28]. Chemical reactions of oxidation and polymerization cause the formation of chalcones and interaction with other compounds present in the food matrix, such as carbohydrates, proteins, fibers, and minerals, which affect the decrease in bioactive compounds as polyphenols in the small intestine [28].

Regarding the undigested samples, it can be observed that the non-impregnated (dry) samples presented high total polyphenols content, due to a single heat treatment (dehydration), unlike the impregnated apples and dried which were subjected to two heat treatments (ohmic heating and dehydration), generating a sensitivity of polyphenols. However, during in vitro digestion, it was observed that total polyphenols content was higher in the impregnated samples due to the phenomenon of electropermeabilization (changes in the membrane) that occur during ohmic heating. This effect causes bioactive compounds to be better retained and protected within the cell, which finally suggests that they are more available at the time of digestion [1].

### 3.3. Changes in the Content of Polyphenols (Monomers) during Digestion In Vitro

**Figure 3:**
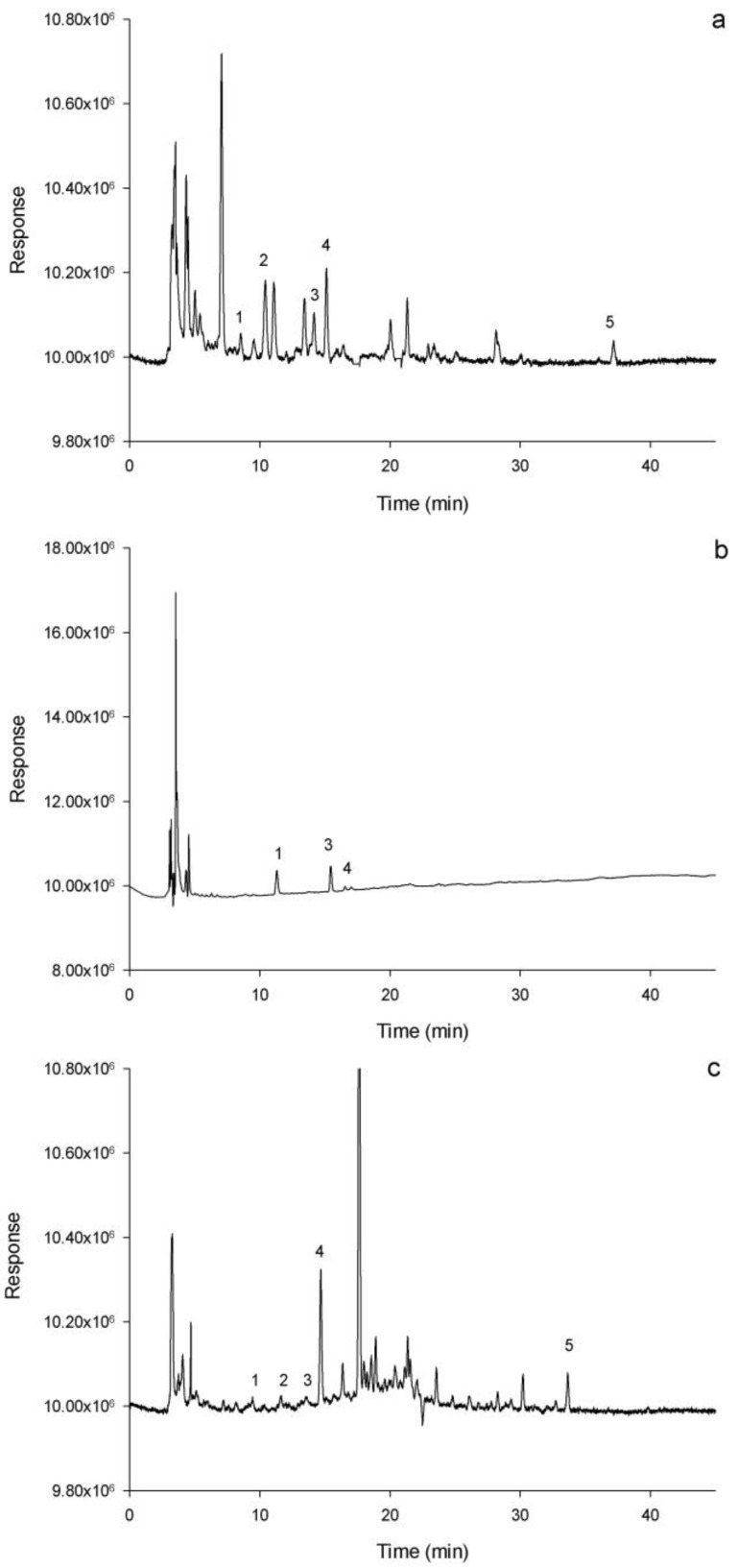
Chromatograms of samples. (**a**) grape juice, (**b**) fresh apple, and (**c**) impregnated apple and dried (VI/OH 50 °C). Peaks: 1 catechin, 2 epigallocatechin, 3 epicatechin, 4 cyanidin and 5 quercetin.

Table 1 shows the total polyphenol content (TPC) and monomeric polyphenol content of fresh/dried apple and the apple impregnated with grape juice. For the monomeric polyphenols present and identified in the samples, the following retention times were obtained: 8.50, 11.70, 14.40, 14.80, and 36.70 min for catechin, epigallocatechin, epicatechin, cyanidin, and quercetin, respectively. As observed in Table 1, in the VI/OH (50 °C) and VI/OH and dried (60 °C) samples, they show an increase in catechin concentration concerning the fresh sample epicatechin and epigallocatechin. However, in the digestive process, these compounds decrease as the in vitro digestion process takes place. As for cyanidin, there is a decrease compared to the fresh sample for these same treatments; possibly, this compound is affected by the thermal conditions of the applied processes. Regarding epigallocatechin, its presence in the VI/OH (50 °C) and VI/OH and dried (60 °C) samples is due to the incorporation of grape juice into the matrix since it is not a phenolic compound present in the apple. As can be seen in Figure 3a–c, the incorporation of epigallocatechin from the grape juice to the matrix is evident.

### 3.4. Antioxidant Capacity of the Snack during In Vitro Digestion

The results presented in Table 2 show the enriched apple snacks’ antioxidant capacity before in vitro digestion (apple snack enriched with grape), comparing the antioxidant capacity methods of the DPPH *β-*Carotene bleaching assay and FRAP assay. The samples treated with VI/OH enriched with grape juice exhibited significantly higher antioxidant activity than the control sample (fresh/dried), probably due to a significantly increased content of bioactive compounds due to the electroporation phenomenon during the ohmic heating treatment [1].

Table 3, Table 4 and Table 5 show gastrointestinal digestion’s influence on enriched apple snacks’ antioxidant activity. The antioxidant capacity was assessed with different methods, namely one method that evaluates sample reducing capacity (FRAP assay), one method that evaluates the radical scavenging capacity (DPPH), and one method that evaluates the ability to inhibit lipid peroxidation (*β-*Carotene bleaching assay). During in vitro digestion, antioxidant compounds could be chemically altered with the consequent modification of their chemical properties and functions, leading to different antioxidant activity results. For the evaluation, antioxidant capacity measurement by more than one method is recommended [29,30]. The digested sample supernatants with the highest antioxidant activity were obtained after in vitro digestion compared to the undigested samples.

In the DPPH radical scavenging activity assay, the highest antioxidant activity obtained from samples enriched with grape juice was 417.20 ± 16.15 µg/mL at the final intestinal phase. The antioxidant capacity of enriched apple snacks (grape juice) increased ≈ 36% after the mouth phase until in vitro digestion ended.

According to *β-*Carotene bleaching assay, the antioxidant capacity of enriched apple snacks (grape juice) increased ≈ 118% after the middle intestinal phase and remained constant until the end of the in vitro digestion (76.20 ± 2.04 %). On the other hand, the antioxidant capacity of control (fresh/dried) increased ≈ 106% after the middle intestinal phase and decreased at the end of the in vitro digestion.

In what concerns ferric reducing antioxidant power (FRAP) assay, results showed that both apple snacks, control (fresh/dried) and enriched (Grape juice), increased the antioxidant capacity at the end of the in vitro digestion. The highest antioxidant activity obtained from samples enriched with grape juice was 288.33 ± 5.5 µM/g at the initial intestinal phase.

## 4. Discussion

### 4.1. Impregnation Kinetics of Polyphenols with Grape Juice

Impregnation kinetics of polyphenols with grape juice was carried out at three temperatures (30, 40, and 50 °C) most significant impregnation peak for the total polyphenol content was at 60 min, to then record a decrease of these due possibly to its degradation by being exposed to the action of the temperature. Some factors favor the impregnation of a compound, such as the high solubility and low viscosity of the solution and the samples’ porosity [31]. De Lima et al. [32] studied different treatments to impregnate calcium in pineapples, finding no significant difference in those samples impregnated only with a vacuum pulse or several vacuum pulses. This behavior is probably due to the polyphenols’ degradation after longer exposure times to the impregnation methods. Some of the factors that favor the impregnation of the compounds into a plant matrix are the high solubility and low viscosity of the impregnation solution and the matrix’s porosity [31].

### 4.2. Total Polyphenol Content during In Vitro Digestion

The polyphenol reduction in the impregnated sample could be due to the degradation of these bioactive compounds during the drying process [33] since they were exposed to dry air for 390 min, while the control sample (fresh/dried apple snack) was dried for 340 min. Nonetheless, it should be noted that samples were dried until equilibrium moisture was reached and not by drying time, obtaining the following values: 10.75% and 10.66% moisture for the fresh/dried and impregnated samples, respectively. The content of total polyphenols in the fresh/dried apple snack was similar to that reported by Quitral et al. [34] in a similar product.

For the apple snack (fresh/dried), there were no significant differences during in vitro digestion from the mouth phase to the initial intestinal phase. Then, upon completing the digestion process, the phenolic content decreased significantly (Figure 2). For the pair of samples, the control, and the enriched apple snack, the phenolic compound concentration throughout the gastric phase was significantly higher than in the other phases of in vitro digestion. This behavior is due to phenolic compound hydrolysis when they are bound to carbohydrates and proteins from the food matrix by enzymatic action and low pH during gastric digestion [27]. In the small intestinal phase (initial, middle, and final intestinal), the phenolic content decreased significantly due to pH changes, from moderately acidic (gastric digestion) to moderately alkaline [9].

### 4.3. Changes in the Content of Polyphenols (Monomers) during Digestion In Vitro

Catechin and quercetin presented significantly higher values in the gastric phase than in the other digestive phases; this is possibly due to the molecules’ stability against neutral or basic pH [35]. The trends in monomeric polyphenols’ behavior during in vitro digestion for the VI/OH and dried treatment (60 ° C) was downward at the end of the digestive process, mainly in quercetin, which decreased by 49.38% concerning the concentration. Initially, before in vitro digestion, the cyanin, catechin, epicatechin, and epigallocatechin decreased by 26.66%, 20.71%, 23.38%, and 21.73%, respectively. This same behavior was reported by Lingua et al. [36] in the digestion of red grapes. However, in the gastric stage, there was a slight increase in epicatechin and quercetin, possibly due to the stomach’s gastric conditions that provoked the compounds’ release.

### 4.4. Antioxidant Capacity of the Snack during In Vitro Digestion

The study of the antioxidant capacity of the compounds from the diet to gastrointestinal digestion is crucial to evaluate their potential effects on human |health. Possible interactions between bioactive compounds and/or food constituents and the effects of luminal factors (including pH and enzymes), food preparation, and food matrix nature could modify bioactive compounds’ chemical structure affecting their bioactivity and their possible beneficial effects [37,38].

The obtained results suggest that the antioxidant components present in apple snacks are stable to pH changes and enzymatic destruction. A possible explanation for the increase in antioxidant capacity can be attributed to thermal treatment, which probably altered the juice composition and caused alterations in the apple slices’ cell walls, thus allowing a more significant release of bioactive compounds from the impregnated snack [9]. Moreover, the radical scavenging activity of polyphenols is strongly pH-dependent, increasing significantly at low pH values. This increase in radical scavenging activity has been attributed to the hydroxyl moieties’ deprotonation present on the polyphenols’ aromatic rings [39].

Liu et al. [40] examined the effects of extrusion on the properties of apple pomace. The antioxidant activity in supernatants from in vitro digestion of extruded apple pomace powder was significantly higher than that of freeze-dried apple pomace. Many factors could account for this increase in antioxidant activity. There was a contribution from the phenolic compounds themselves due to their release and conversion reactions. Ryan et al. [41] verified that red-colored juices showed high antioxidant capacity, and this capacity was further enhanced following an in vitro digestion procedure.

## 5. Conclusions

Through the combination of VI and OH, an apple snack enriched with grape juice was created. It was possible to incorporate polyphenols into the food matrix (apple slices), which increased its antioxidant capacity. During the in vitro digestion procedure, the highest total polyphenol content was identified in the gastric phase for apple snacks enriched with grape juice. In vitro digestion demonstrated that polyphenols in enriched apple snacks remained bioaccessible during thermal treatment (VI/OH). The tendencies in monomeric polyphenols’ behavior during in vitro digestion for the VI/OH and dried treatment (60 °C) was descending at the end of the digestive process, mainly in quercetin, which decreased by 49.38% concerning the intimal concentration before digestion. As for the samples enriched with grape juice, epigallocatechin was incorporated from the juice into the matrix. In the samples enriched with grape, most of the monomeric polyphenols decreased across the different gastric phases. Likewise, the decrease in these monomeric compounds agrees with the total polyphenol content.

Regarding the antioxidant capacity of the samples impregnated with IV/OH 50 °C treatment (dried 60 °C), their antioxidant potential increased during the stages of the digestive process. The previous results indicate that the combination of vacuum impregnation and ohmic heating is an excellent technological option to obtain products that allow for the incorporation of antioxidants and maintain nutritional value through various processes. However, further studies need to be carried out with in vivo models to validate and compare the results obtained in this investigation.

## Figures and Tables

**Table 1:** Monomeric polyphenols, flavonoids content of fresh (fresh/dried), enriched apple snack, and in vitro digestion of impregnated with grape juice using VI/OH 50 °C and dried 60 °C.

Treatment	TPC (mg GAE/100 g d.m.)			Flavonoids (mg/100 g d.m.)														
				Cyanidin			Catechin			Epicatechin			Quercetin			Epigallocatechin		
Fresh	432.77	±	4.86 ^b^	57.59	±	3.18 ^a^	234.14	±	13.25 ^b^	11.73	±	0.63 ^e^	10.77	±	0.64 ^a^	ND		
Dried apple	382.30	±	10.20 ^c^	1.80	±	0.04 ^f^	5.89	±	0.49 ^gh^	1.47	±	0.05 ^ij^	1.93	±	0.07 ^de^	ND		
Mouth DA	107.40	±	15.54 ^i^	1.95	±	0.10 ^ef^	4.15	±	0.11 ^h^	2.48	±	0.13 ^hi^	1.90	±	0.99 ^e^	ND		
Gastric DA	120.99	±	21.18 ^i^	1.60	±	0.06 ^f^	3.08	±	0.16 ^h^	3.87	±	0.16 ^h^	1.84	±	0.15 ^e^	ND		
Initial int. DA	117.83	±	21.99 ^i^	2.57	±	0.16 ^def^	2.76	±	0.12 ^h^	2.44	±	0.15 ^hi^	2.02	±	0.05 ^de^	ND		
Meddle DA	115.71	±	19.59 ^i^	2.94	±	0.13 ^def^	2.03	±	0.12 ^h^	0.83	±	0.03 ^j^	2.08	±	0.08 ^de^	ND		
Final Int. DA	103.65	±	24.56 ^i^	2.39	±	0.18 ^def^	2.49	±	0.15 ^h^	1.93	±	0.11 ^ij^	2.51	±	0.21 ^d^	ND		
VI/OH (50 °C)	720.26	±	4.61 ^a^	13.95	±	0.90 ^b^	300.95	±	12.05 ^a^	47.65	±	2.98 ^a^	8.73	±	0.54 ^b^	22.21	±	1.41 ^a^
VI/OH & dried (60 °C)	322.29	±	10.60 ^d^	10.39	±	0.52 ^c^	62.13	±	4.11 ^c^	25.14	±	1.56 ^b^	4.09	±	0.10 ^c^	16.24	±	0.61 ^b^
Mouth VD	245.68	±	9.78 ^fg^	8.99	±	0.25 ^c^	36.81	±	2.84 ^d^	15.53	±	0.58 ^d^	1.96	±	0.10 ^de^	12.88	±	0.32 ^c^
Gastric VD	271.85	±	7.64 ^e^	3.55	±	0.20 ^d^	24.65	±	1.03 ^e^	17.48	±	0.37 ^c^	2.07	±	0.05 ^de^	10.37	±	0.67 ^d^
Initial int. VD	263.50	±	4.27 ^ef^	3.32	±	0.12 ^de^	16.13	±	0.91 ^fg^	10.15	±	0.21 ^ef^	1.79	±	0.07 ^e^	4.08	±	0.30 ^e^
Middle Int. VD	235.20	±	6.76 ^g^	3.55	±	0.19 ^d^	13.55	±	0.48 ^fg^	9.32	±	0.27 ^f^	1.95	±	0.03 ^de^	3.59	±	0.27 ^e^
Final Int. VD	187.85	±	9.16 ^h^	2.77	±	0.13 ^def^	12.87	±	0.64 ^f^	5.88	±	0.39 ^g^	2.02	±	0.04 ^de^	3.53	±	0.10 ^e^

^a–j^ when significant difference (*p* ≤ 0.05) between columns according to LSD test is identified by lower case letters in the superscript. d.m.: dry matter. DA: dried apple; VD: VI/OH & dried. ND: Not Detected.

**Table 2:** Antioxidant capacity DPPH, *β-*Carotene bleaching, and Ferric reducing antioxidant power (FRAP) assay of dehydrated apple (control fresh/dried) and Enriched apple snack (grape juice) undigested samples.

	DPPH EC_50_ (µg/mL)			*β-*Carotene Bleaching AA%			FRAP AA TE(µM/g)		
Control (fresh/dried)	680.04	±	3.62 ^a^	10.11	±	1.02 ^b^	170.78	±	1.92 ^b^
Enriched apple Snack (Grape Juice)	538.77	±	4.78 ^b^	26.55	±	4.00 ^a^	368.56	±	19.53 ^a^

^a, b^ when significant difference (*p* ≤ 0.05) between rows according to LSD test are identified by lower case letters in the superscript.

**Table 3:** Antioxidant capacity DPPH assay of dehydrated apple (control fresh/dried) and Enriched apple snack (grape juice) during in vitro digestion.

	Control (Fresh/Dried) EC_50_ [µg/mL]			Enriched Apple Snack (Grape Juice) EC_50_ [µg/mL]		
Phases						
Mouth	415.13	±	1.65 ^bC^	654.10	±	7.17 ^aB^
Gastric	681.39	±	7.25 ^aA^	684.47	±	10.58 ^aA^
Initial Int.	417.47	±	8.45 ^bC^	522.47	±	11.11 ^aC^
Middle Int.	421.53	±	12.99 ^bC^	449.60	±	5.82 ^aD^
Final Int.	441.58	±	13.87 ^aB^	417.20	±	16.15 ^aB^

^a, b, c…^ Different lowercase letters in a column indicate statistical difference (*p* ≤ 0.05) according to LSD test. ^A, B, C...^ Different capital letters in a row indicate statistical difference (*p* ≤ 0.05) according to LSD test.

**Table 4:** *β-*Carotene bleaching assay of dehydrated apple (control fresh/dried) and Enriched apple snack (grape juice), during in vitro digestion.

	Control (Fresh/Dried) AA%			Enriched Apple Snack (Grape Juice) AA%		
Phases						
Mouth	10.68	±	0.24 ^bC^	34.87	±	1.36 ^aC^
Gastric	9.21	±	0.43 ^bD^	41.84	±	2.00 ^aB^
Initial Int.	10.85	±	0.37 ^bC^	36.01	±	1.74 ^aC^
Middle Int.	22.10	±	0.37 ^bA^	76.11	±	2.94 ^aA^
Final Int.	18.92	±	0.86 ^bB^	76.20	±	2.04 ^aA^

^a, b, c…^ Different lowercase letters in a column indicate statistical difference (*p* ≤ 0.05) according to LSD test. ^A, B, C...^ Different capital letters in a row indicate statistical difference (*p* ≤ 0.05) according to LSD test.

**Table 5:** Ferric reducing antioxidant power (FRAP) assay of dehydrated apple (control fresh/dried) and Enriched apple snack (grape juice) during in vitro digestion.

	Control (Fresh/Dried) AA TE(µM/g)			Enriched Apple Snack (Grape Juice) AA TE(µM/g)		
Phases						
Mouth	56.85	±	3.21 ^bE^	155.00	±	5.56 ^aC^
Gastric	105.00	±	5.56 ^bD^	162.41	±	3.21 ^aC^
Initial Int.	143.89	±	5.56 ^bC^	288.33	±	5.56 ^aA^
Middle Int.	225.37	±	3.21 ^bB^	273.52	±	13.98 ^aAB^
Final Int.	258.70	±	6.42 ^aA^	266.11	±	9.62 ^aB^

^a, b, c…^ Different lowercase letters in a column indicate statistical difference (*p* ≤ 0.05) according to LSD test. ^A, B, C...^ Different capital letters in a row indicate statistical difference (*p* ≤ 0.05) according to LSD test.
